# Major prospects for exploring canine vector borne diseases and novel intervention methods using 'omic technologies

**DOI:** 10.1186/1756-3305-4-53

**Published:** 2011-04-13

**Authors:** Robin B Gasser, Cinzia Cantacessi, Bronwyn E Campbell, Andreas Hofmann, Domenico Otranto

**Affiliations:** 1 Department of Veterinary Science, The University of Melbourne, 250 Princes Highway, Werribee, Victoria 3030, Australia; 2 Structural Chemistry Program, Eskitis Institute for Cell & Molecular Therapies, Griffith University, Brisbane, Queensland, Australia; 3 Dipartimento di Sanità Pubblica e Zootecnia, Facoltà di Medicina Veterinaria, Università di Bari, Str. prov. le per Casamassima Km 3, 70010, Valenzano, Bari, Italy

## Abstract

Canine vector-borne diseases (CVBDs) are of major socioeconomic importance worldwide. Although many studies have provided insights into CVBDs, there has been limited exploration of fundamental molecular aspects of most pathogens, their vectors, pathogen-host relationships and disease and drug resistance using advanced, 'omic technologies. The aim of the present article is to take a prospective view of the impact that next-generation, 'omics technologies could have, with an emphasis on describing the principles of transcriptomic/genomic sequencing as well as bioinformatic technologies and their implications in both fundamental and applied areas of CVBD research. Tackling key biological questions employing these technologies will provide a 'systems biology' context and could lead to radically new intervention and management strategies against CVBDs.

## Background

Although difficult to estimate, the impact of canine vector-borne diseases (CVBDs) to dog and human populations is substantial [1-3]. Particularly bacteria (e.g., species of *Rickettsia*, *Ehrlichia *and *Borrelia*), protists (including species of *Babesia*, *Leishmania *and *Trypanosoma*), nematodes (e.g., species of *Dirofilaria *and *Acanthocheilonema*), and their vectors (including mosquitoes, fleas, ticks and/or sand flies) constitute major components of the burden of CVBDs [2-4]. With a changing global climate, in the absence of effective preventative approaches and new intervention strategies, the disease burden linked to many neglected CVBDs is likely to increase further [5]. In spite of advances made, there are still major knowledge gaps in CVBDs. These gaps exist mainly in the fundamental molecular biology, epidemiology, ecology and population genetics of causative agents and their vectors, emerging drug resistance issues as well as infection processes and virulence factors [1]. Moreover, substantial limitations in diagnosis and intervention also represent critical obstacles to the effective control of CVBDs. Although sustained research and funding have contributed significantly to an improved understanding of human vector-borne diseases, such as malaria and trypanosomiasis, this is not the case for many parasitic diseases, which are neglected in terms of research and development [6]. A fundamental change is needed, particularly in relation to CVBDs.

The 'omics era has brought about substantial prospects for investigating some important pathogens and their vectors, providing insights into their epidemiology, ecology, evolution and cellular processes. Available genomes are considered to represent crucial infrastructure for elucidating novel avenues to tackle infectious diseases. However, the relatively high cost and laborious nature of molecular and biochemical research has sometimes been an impediment to progress. Revolutionary developments in a range of 'omic (e.g., genomic, proteomic, metabolomic, glycomic and lipidomic) technologies [7] now provide unprecedented opportunities to explore CVBDs on a scale and at a rate that was unimaginable just a couple of years ago, providing major opportunities for addressing critically important areas of research for the first time ever. Future research should harness such technologies to address major knowledge gaps for CVBDs. Elucidating the pathogens, their relationship with their vector(s) and definitive hosts, the disease(s) as well as the epidemiology and ecology of pathogens causing CVBDs will have substantial prospects to improve the treatment, prevention and control of these parasites in years to come. The intent of this presentation is not to review the literature on CVBDs, rather to take a prospective view of the impact that 'omics technologies could have on CVBD research. The emphasis has been placed on describing the principles of transcriptomic/genomic sequencing as well as bioinformatic technologies and their implications in both fundamental and applied areas.

Transcriptomics, for example, is the molecular science of examining, simultaneously, the transcription of all genes at the level of the cell, tissue and/or whole organism, allowing inferences regarding cellular functions and mechanisms. The ability to measure the transcription of thousands of genes simultaneously has led to advances in all biomedical fields, from understanding the basic function in model organisms, such as the yeast, *Saccharomyces cerevisiae *and the vinegar fly, *Drosophila melanogaster *[8-10], to studying molecular processes or mechanisms associated with growth, development and reproduction, to the exploration of the mechanisms of survival and drug-resistance. For more than a decade, transcriptomes have been determined by sequencing expressed sequence tags (ESTs), mainly using a conventional (Sanger) approach [11,12], whereas levels of transcription have been established quantitatively or semi-quantitatively by real-time PCR [13] and/or cDNA microarrays [14]. The use of such technologies has been accompanied by an increasing demand for analytical computer tools for the efficient annotation of nucleotide sequence datasets, particularly within the framework of large-scale EST projects [15]. With a substantial expansion of nucleic acid sequencing has come the development of algorithms for sequence assembly, analysis and annotation, in the form of individual programs [16-18] and integrated pipelines [19,20], some of which have been accessible *via *the worldwide web [19,21,22]. However, the cost and time associated with conventional sequencing and/or the design of customized analytical tools have driven the search for alternative and improved methods [23].

## Next-generation sequencing technologies

There has been an explosion in next-generation sequencing (NGS) technologies [24-27], which now provide unprecedented opportunities to explore *de novo *the transcriptomes and genomes of different species and developmental stages of pathogens, their vectors and/or their definitive hosts. Although introduced recently, the capacity of such techniques to generate millions to hundreds of millions of sequences in parallel has placed them at the forefront of the molecular research [28-30]. Currently available NGS sequencing platforms include 454/Roche [24], Illumina/HiSeq [25] and SOLiD [26].

The 454/Roche platform [24] employs a sequencing-by-synthesis approach, by which cDNA is randomly fragmented (by 'nebulization') into 500-1000 base pair (bp) fragments. For the preparation of the library, an adaptor is ligated to each end of these fragments, which are then mixed with a population of agarose beads whose surfaces anchor oligonucleotides complementary to the 454-specific adapter sequence, such that each bead is associated with a single fragment. Each of these complexes is transferred into individual oil-water micelles containing amplification reagents and is then subjected to an emulsion PCR (emPCR) step, during which ~10 million copies of each cDNA are produced and bound to individual beads. In the sequencing phase, the beads anchoring the cDNAs are deposited on a pico-titre plate, together with other enzymes required for the pyrophosphate sequencing reaction (i.e., ATP sulfurylase and luciferase). The sequencing is carried out by flowing sequencing reagents (nucleotide and buffers) over a plate [31]. To date, the 454 sequencing technology is a 'long-read' (100-600 bp) platform and is often used for *de novo *genomic or transcriptomic studies.

The Illumina/HiSeq (formerly Solexa) technology has features that differ significantly from the 454 approach [25]. After fragmentation of cDNA sample into a shotgun library, Illumina-specific adaptors are ligated *in vitro *to each cDNA template; one terminus of the template is covalently attached to the surface of a glass slide (or flow cell). Attached to the flow cell are primers complementary to the other end of the template, which bend the cDNAs to form bridge-like structures. During the amplification step (bridge-PCR), clonal clusters, each consisting of ~1000 amplicons, are generated and immobilized to a single physical location on the slide. Subsequently, the cDNAs are linearised, and the sequencing reagents are directly added to the flow cell, with four fluorescently labelled nucleotides. After the incorporation of individual fluorescent bases, the flow cell is interrogated with a laser in several locations, which results in several image acquisitions at the end of a single synthesis cycle [31]. This technology is considered ideal for re-sequencing projects, targeted sequencing, single nucleotide polymorphism (SNP) analyses and gene transcription studies.

The SOLiD platform employs the enzyme DNA ligase, instead of a polymerase [26]. Briefly, after an emPCR step, the adaptor sequences of the cDNA templates bind to complementary primers that are covalently anchored to a glass slide. Subsequently, a set of four fluorescently labelled di-probes (octamers of random sequence, except known dinucleotides at the 3'-terminus) is added to the sequencing reaction. In case an octamer is complementary to the template, it is ligated, and the two specific nucleotides can be called; subsequently, an image is acquired and the fluorescent dye is removed, so that other octamers can be ligated. After multiple ligations (e.g., 7 ligations for a 35 bp read), the newly synthesized cDNA is removed and the primer is inactivated. This process is repeated multiple times from different starting points of the cDNA templates, so that each position is sequenced at least twice ('two-base-calling'). Because of the short read-length, the range of applications of the SOLiD system is considered similar to that of the Illumina technology and includes (targeted) re-sequencing projects, SNP detection and gene transcription studies.

In the last years, a range of studies have demonstrated the utility of NGS technologies for investigating, for instance, aspects of the molecular biology, systematics and population genetics of parasites [32-36]. In particular, 454 technology was used recently for the rapid *de novo *sequencing of the transcriptomes of numerous pathogens of humans and animals [33-41], yielding substantial datasets and providing a significant step forward. The development of practical and efficient bioinformatic tools has now become crucial for comprehensive analyses of such datasets.

## Bioinformatic tools for the analysis of sequence datasets

The application of NGS technologies has been accompanied by an expansion of bioinformatic tools for the analysis of DNA, RNA and protein sequence datasets. This expansion has resulted in the development of a number of programs and/or integrated pipelines accessible via the world-wide web (www) (e.g., [16,19,21,42,43]). The principles, methods and protocols for the analysis of sequence data, together with currently available bioinformatic tools and pipelines, have been reviewed [42].

In brief, following the acquisition of data, sequences are firstly screened for repeats, contaminants and/or adaptor sequences [42,44] and 'clustered' (= assembled) into contiguous sequences (of maximum length; called contigs) based on sequence similarity (see [42]). Long- (e.g., generated by Sanger sequencing, 454 platform) and short-reads (e.g., Illumina and SOLiD platforms) are assembled using the algorithms 'overlap-layout-consensus' [45] and 'de Bruijn graph' [46,47], respectively. For the former algorithm [45], pair-wise overlaps among reads are computed and stored in a graph; all graphs are then used to compute a layout of reads and a consensus sequence of contigs [21,48-53]. For the 'de Bruijn graph' [46,47], reads are fragmented into short segments, denominated 'k-mers', where 'k' represents the number of nucleotides in each segment; overlaps between or among k-mers are captured and stored in graphs, which are subsequently used to generate the consensus sequences [47,52-56].

Following assembly, the contigs and single reads (or singletons) are compared, using different types of the Basic Local Alignment Software Tool (BLAST; [57]) with known sequence data available in public databases, in order to assign a predicted identity to each query sequence if significant matches are found [42]. In addition, assembled nucleotide sequences are usually conceptually translated into predicted proteins using algorithms that identify protein-coding regions (ORFs) from individual contigs [22,58,59]. Once peptides are predicted, protein analyses, including amino acid sequence comparisons with data available in public databases, and known protein domains, are then undertaken [17,42,60-63]. Public databases (accessible *via *www) represent comprehensive collections of nucleotide and amino acid sequences, where all publicly available nucleotide sequences are stored and curated [64-66]; in addition, each sequence is stored as a separate record and linked to salient information, such as primary source, references and predicted and/or experimentally verified biological features. For transcriptomic datasets, examples of databases include the UniGene [67] and the Sequence Read Archive (SAR) [68]. In addition to these general databases, there are various specialized collections of gene and protein information on particular (model) organisms about which much is known. Examples include the databases for *S. cerevisiae *(yeast; http://www.yeastgenome.org/) [69], *D. melanogaster *(vinegar fly; http://flybase.org/[70], *Mus musculus *(mouse; http://www.informatics.jax.org/) [71] and *Caenorhabditis elegans *(free-living nematode; WormBase at http://www.wormbase.org) [72,73].

A web-based bioinformatic pipeline (= ESTExplorer) was established for the automated analysis and annotation of nucleic acid datasets (both at the nucleotide and amino acid levels) [19], and shown to substantially accelerate and facilitate the analyses of sequences (generated using conventional Sanger sequencing) compared with traditional database searches [20]. However, sequences generated by NGS are significantly shorter (454/Roche: ~400 bases; Illumina/SOLiD: ~60 bases) than those determined by Sanger sequencing (0.8-1 kb), which poses a significant challenge for assembly. In addition, the data files generated by these technologies are often gigabytes to terabytes (1 × 10^9 ^to 1 × 10^12 ^bytes) in size, substantially increasing the demands placed on data transfer and storage, such that most web-based interfaces are no longer suited for large-scale analyses. In order to overcome this limitation, a recent report [39] described the development of an integrated bioinformatic workflow system for the analysis and annotation of large sequence datasets produced by NGS, in which the majority of the software was derived from existing application tools (e.g., CAP3; [21]), available as web-based interfaces. These applications, optimized using the Linux operation system, were incorporated into pre-existing scripts (i.e., Perl, Python and Unix shell), and can be downloaded http://research.vet.unimelb.edu.au/gasserlab/index.html[39] and readily executed, also by scientists with limited bioinformatic expertise. This workflow system has proved very useful for time-efficient and accurate analyses of large-scale transcriptomic datasets generated by NGS and for distilling biologically meaningful information (such as predictions of essential molecules) on the parasite, the vector or the host under investigation.

## Exciting prospects in both fundamental and applied areas

Knowledge of the transcriptomes and proteomes of different developmental stages of a parasite, its vector and its definitive host is central to gaining an enhanced understanding of the molecular mechanisms that govern essential biological, infection and disease processes and, ultimately, could assist in identifying possible avenues for the development of novel intervention strategies. Accurate bioinformatic analyses of nucleic acid and protein sequence data (often by comparison with or inference from reference organisms) are crucial, in the absence of information for the organism under study, in providing biological meaningful molecular biological information about CVBDs. Until recently, detailed bioinformatic analyses of such datasets have been restricted largely to specialized laboratories with substantial computer and software capacities. The development of flexible and practical bioinformatic workflow systems is beginning to provide scientists with user-friendly tools for the analysis of massive datasets.

Currently, due to a lack of complete genomic sequences for many pathogens and vectors (and different strains thereof) associated with CVBDs, newly generated sequence datasets need to be assembled *de novo*, which means that pooled reads are assembled without a bias towards known sequences [47]. Due to the amount of RNA required for NGS (~5-10 μg) [74], transcriptomes usually originate from numerous individuals, potentially leading to an increased complexity of the sequence data acquired (linked, for instance, to a biased nucleotide content, single nucleotide polymorphisms [SNPs] and other types of sequence variation) and sometimes posing challenges for the data assembly. In terms of complexity, computational and time requirements, *de novo *assemblies are much slower and more computer-memory intensive than knowledge-based (mapping) assemblies, in which reads are aligned and assembled against an existing reference sequence (representing the same species or genetic variant) [18]. In addition, reliable *de novo *assemblies are highly dependent upon the availability of long reads (>100 bases) and of high-coverage, paired-end sequence data [75]. In previous studies, the complementary nature of the 454 and Illumina sequencing platforms has allowed the assembly of raw reads into large scaffolds without a need for a reference sequence [76-78].

In the absence of reference genomes for agents and vectors linked to CVBDs, accurate assembly of sequence data is a crucial step in examining coding genes and, ultimately, addressing biological questions regarding gene and protein functions. Functions are initially predicted by 'sequence annotation' (= the process of gathering all available information and relating it to the sequence assembly both by experimental and computational means [79]. Accurate annotation is dependent on the efficiency of the updates and curation. Presently, open-source programs and databases routinely employed for the bioinformatic analyses of sequence data are available *via *multiple portals, thus requiring significant efforts to maintain accurate and up-to-date assembly and annotation pipelines [80]. In addition, the rate at which public databases are updated and corrected varies considerably. For instance, the Swiss-Prot database http://au.expasy.org/sprot/ accepts corrections from its user community, whereas GenBank http://www.ncbi.nlm.nih.gov/genbank/ only accepts corrections from the author of an entry [81], thus significantly affecting the accuracy and speed with which new sequences are annotated. In addition, some information-management systems incorporate data from large-scale projects, but often, the annotation of single records from the literature is slow [82]. Given that, presently, the annotation of sequence data for parasites and vectors relies heavily on the use of bioinformatic approaches and already annotated/curated sequence data for a wide range of organisms, these aspects deserve careful consideration.

The analyses and annotation of large-scale transcriptomic, proteomic and genomic sequence datasets for pathogens could be facilitated through the establishment of a 'reference' website for CVBDs. Such a website could provide regular releases of newly developed and validated bioinformatic pipelines for the analyses of sequence datasets. It could also provide links to regularly updated databases that are routinely employed for the annotation of new sequences as well as a distinct, high-quality database of curated functional annotations, supported by experimental data published in peer-reviewed, international publications. In the future, the establishment of a 'centralized' resource to enable the sharing and optimization of bioinformatic pipelines for sequence processing and annotation and, more broadly, to allow access to new sequence data, and experimental protocols and relevant literature would be advantageous.

The annotation of peptides inferred from a dataset is conducted by assigning predicted biological function/s based on comparison with existing information available for related organisms in public databases, including InterPro http://www.ebi.ac.uk/interpro/, Gene Ontology, http://www.geneontology.org/, OrthoMCL http://www.orthomcl.org/, BRENDA http://www.brenda-enzymes.org/. Using this approach, predictions for key groups of molecules can be made regarding their fundamental functional and essential roles in biological processes [61]. Such groups include molecules linked to the physiology of the nervous system [37], the formation of the cuticle (arthropods and nematodes) [37,83], reproduction, development, signal transduction and/or pathogen invasion and disease processes (e.g., proteases and protease inhibitors, protein kinases and phosphatases) [36-39,84].

The bioinformatic prediction and prioritization of novel drug targets involves 'filtering' [85,86] and usually includes inferring targets based on key principles and requirements [87-91]. First, target proteins should have one or more essential roles in fundamental biological processes of the pathogen and/or vector, such that the disruption of the molecule or its gene will damage and/or kill both or either and thus disrupt disease transmission or disease itself, but not affect the host [90,92]. In the absence of phenotypic data for many pathogens/vectors, the prediction of drug target candidates in eukaryotic pathogens/vectors can be assisted by using extensive information on function and essentiality in a range of eukaryotic organisms, including *S. cerevisiae*, *D. melanogaster*, *C. elegans *and *M. musculus*. This information can be accessed *via *public databases, including FlyBase at http://flybase.org/, WormBase at http://www.wormbase.org, Mouse Genome Informatics at http://www.informatics.jax.org/ and *Saccharomyces *Genome Database at http://www.yeastgenome.org/) [39,89,93-95]. Since most effective drugs achieve their activity by competing with endogenous small molecules for a binding site on a target protein [96], the amino acid sequences predicted from essential genes should be screened for the presence of relatively conserved ligand-binding domains [96,97]. Lists of inhibitors, known based on experimental evidence, to specifically bind to such domains, can be compiled. However, the predictions made are intended to support hypothesis-driven or applied research and thus require extensive experimental investigations. The main advantage for a number of CVBD pathogens (e.g., *Babesia *and *Leishmania*) over, for example, some parasitic helminths, is that they can be propagated readily *in vitro *(e.g., [98,99]). This provides unique prospects to test gene function(s) by double-stranded RNA interference, transgenesis and/or deletion studies as well as using small molecular inhibitors (cf. [100-102]).

Based on recent evidence [103-105], guanosine triphosphatases (GTPases), protein phosphatases and protein kinases seem to represent attractive drug target candidates for a range of pathogens, but have not yet been examined on a genome-wide scale and in a systematic manner for most CVBDs. Multiple cellular signaling pathways function through the activity of small GTP-binding proteins to regulate multiple biological processes, such as transmembrane signal transduction, cytoskeletal reorganization, gene expression, intracellular vesicle trafficking, microtubule organization and nucleocytoplasmic transport [106]. GTPases are small (~20-28 kDa), monomeric proteins belonging to six families (i.e., Ras, Rho, Rab, Arf, Ran and Rad; [107]). These regulatory proteins act as bi-molecular switches that cycle between two conformational states (i.e., GDP-bound ["inactive" state] and GTP-bound ["active" state]) and hydrolyze GTP. In humans, the aberrant regulation of GTPases is linked to a number of dysfunctions, including neurological and developmental disorders and cancer [108]. In addition, intracellular pathogenic bacteria, such as *Mycobacterium tuberculosis*, are known to target host GTPases to evade host immune responses to facilitate the infection process [109]. Such information has stimulated efforts to develop novel therapeutic strategies to inhibit the function of GTPases. For instance, treatments with farnesyltransferase inhibitors, to block the oncogenic properties of Ras GTPases, have been shown to be effective in significantly reducing the progression of various forms of cancer, including carcinomas of the colon, pancreas and lung, neurofibrosarcoma and chronic myelogenous leukaemia, in experimental animals [110,111] and the migration and organization of the cytoskeleton of human prostate cancer cells [112]. Although the overall structure of individual small GTPases is conserved across eukaryotes, the filtering of datasets for the organism of interest (i.e., pathogen and/or vector) allows the identification of significant differences in sequence of GTPases between the invertebrate and the definitive host. These differences might be considered in future studies, aimed at assessing the possibility of designing and synthesizing selective and specific inhibitors against parasite GTPases. Homology modelling [113,114], X-ray crystallography/nuclear magnetic resonance (NMR) and docking [115-120] studies should assist in this process.

Selected protein kinases (PKs) are also potential drug targets for a range of pathogens. PKs belong to a large family of proteins regulating development, cell division, differentiation and metabolism in many organisms; these molecules are considered the second most important group of drug targets after GPCRs [121,122]. The family of PKs comprises cell surface receptors and non-receptor or cytosolic kinases. Integrated genomic-bioinformatic-chemoinformatic approaches have been employed for the identification and screening effective PK inhibitors as therapeutic agents [123-125]. For example, in studies aimed at identifying novel inhibitors of a human tyrosine kinase involved in the development and progression of chronic myelogenous leukemia, 15 compounds were selected following *in silico *screening of a database of 200,000 known inhibitors [124]. Of these compounds, eight were shown to selectively inhibit the growth of leukemia *in vitro *[124]. In another study, novel and selective inhibitors of caseine kinase II (CK2) were identified *via in silico *screening of a database containing ~400,000 compounds, followed by *in silico *docking [125]. These examples indicate the advantages of using computer-aided tools for the rational prediction and design of drugs for subsequent *in vitro *and *in vivo *efficacy testing [126]. Nonetheless, it is clear that any compound shown to be efficacious must also be rigorously tested for its safety (see [127]; http://www.ich.org/cache/compo/276-254-1.html).

Because of the regulatory role that PKs play in a number of signaling pathways in the cell, interference with their activity can result in the disruption of fundamental homeostatic processes in parasites [105]. In the last years, protein kinases have received particular attention as drug targets in protists, such as species of *Plasmodium*, *Leishmania *and *Trypanosoma *and helminths [105]. For instance, particular inhibitors of pyrrole and imidazopyridine cyclic guanosine monosphosphate-dependent protein kinases of *Leishmania major *have been shown to severely impair the growth of the promastigote forms of this protozoan parasite *in vitro *[128]. In some helminths, for example, PK inhibitors (i.e., tyrphostins AG1024 and AG538) have been shown to significantly affect the survival and development of the adult parasite through the blockage of glucose uptake [122]. The inactivation of PKs with herbimicin A has also been shown to interfere with mitosis, thus significantly affecting the expression of proteins essential for egg production in the worm [129]. Although the crystal structures of PKs in many pathogens have not yet been defined, progress has been made in the identification and design of effective inhibitors based on homology models for protein kinases from humans [105]. There is evidence that the active sites of parasite PKs display subtle differences compared with their human counterparts [105], which is considered promising for the development of parasite-specific kinase inhibitors. However, much more study is required to establish the potential of PK inhibitors against pathogens causing CVBDs. This is obviously a research area worth pursuing.

## Concluding remarks

Vector-borne diseases, of which CVBDs represent a substantial component, represent ~17% of the burden of all infectious diseases and have a major socioeconomic impact worldwide [130]. In addition to their veterinary importance, some CVBD-causing agents are of major zoonotic importance. Although various studies have provided improved insights into the epidemiology of CVBDs using molecular methods, there has been limited study of fundamental molecular aspects of many pathogens, their vectors, pathogen-host relationships and disease as well as drug or insecticide resistance using some of the advanced 'omic technologies described here. Tackling fundamental biological questions using these technologies and understanding the relationship among pathogens/vectors/environment will provide a 'systems biological context' to better understand CVBDs and their epidemiology and should lead to the design of radically new intervention and management strategies against these diseases.

For instance, from a fundamental perspective, genomic sequencing and the definition of a wide range of genetic markers for use in specific and sensitive diagnostic tools could provide a solid foundation for addressing questions regarding the complex network of biological and ecological factors involved in pathogen/host/environment interactions and the immunological idiosyncrasies of receptive hosts in endemic regions as well as the role of asymptomatic, chronically infected animals and those infected with multiple pathogens [2]. In this context, using well-defined genetic and transcriptomic tools, it would be interesting to address the question as to whether simultaneous infections with multiple vector-borne pathogens (compared with a single infection) induce synergistic and pronounced immunosuppression in infected animals. Moreover, the application of -omics tools could also assist in comprehensively studying the complex intracellular pathways that are manipulated or regulated by one or multiple pathogens (e.g., species of *Leishmania *and *Ehrlichia*) to evade the immune response of the host and significantly complicating the progression and expression of disease in individual patients (cf. [131,132]). It would also be very useful to investigate the resistance and susceptibility of, for examples, particular dog breeds to CVBD-agents and their vectors. For instance, a genomic comparison between Ibizan hounds (which are resistant to leishmaniasis; [133]) and other breeds, such as Boxers, as well as transcriptomic/proteomic comparisons of the responses of these dogs to infection and disease would be very interesting. In a broader context, gaining improved insights into the relationship between host genotype (through genomic sequencing) and phenotype (degree of disease expression) in response to particular CVBD-pathogens and/or intervention approach (e.g., treatment/vaccination) would be particularly informative and could assist in a deeper understanding the genetic basis of disease. From an epidemiological perspective, also changes in the spatial and temporal distribution of pathogens, vectors and/or their hosts, as a result of climatic change and global warming, might also be monitored using metagenomic approaches. These examples indicate clearly that there are many exciting fundamental areas to tackle using genomic, proteomic and immunomic tools in the very near future.

From an applied perspective, clearly, the improved prediction and prioritization of drug and vaccine targets in CVBD pathogens or repellants against vectors is a key area. NGS will provide the efficiency and depth-of-coverage required to rapidly define *de novo *the complete genomes of hosts, CVBD pathogens and their vectors. Repertoires of drug or vaccine targets could be inferred on a global scale. For example, the parasite kinome (= the complete set of kinase genes in the genome) could represent a unique opportunity for the design of pathogen-selective inhibitors [105] for subsequent validation by high throughput screening of parasites [134-137]. The combined use of genomic, transcriptomic, proteomic and metabolomic datasets will be crucial to identifying other groups of molecules essential to the development and survival of a pathogen for the design of novel classes of small molecular inhibitors. Clearly, an integrated use of 'omic technologies will pave the way to a better understanding of the systems biology of CVBDs on a scale never before possible, and, hopefully, will provide golden opportunities for the development of entirely new intervention strategies in public-private partnerships.

## Competing interests

The authors declare that they have no competing interests.

## Authors' contributions

RBG, CC, BEC, AH and DO all contributed actively to the drafting of the manuscript.
